# Developing effective workforce training to support the long‐term care of older adults: A review of reviews

**DOI:** 10.1111/hsc.13897

**Published:** 2022-07-05

**Authors:** Louise Newbould, Kritika Samsi, Mark Wilberforce

**Affiliations:** ^1^ Department for Social Policy and Social Work University of York York UK; ^2^ King's College London London UK

**Keywords:** geriatric, learning framework, older adults, review, staff training, workforce development

## Abstract

This review of reviews aimed to identify and synthesise evidence to support the design of learning interventions for non‐registered practitioners supporting older people in long‐term care (people's own homes, hospices or residential/nursing care). Our objectives were to inform the analysis part of the Analysis, Design, Development Implementation and Evaluation framework by finding evidence on the following five components of learning: content, format (teaching strategies and resources/media), structure, contextual factors (barriers and enablers) and measures used when monitoring the effectives of learning. Databases searched included Pro‐quest (ASSIA), Scopus, Ovid (PsycINFO, Medline, Embase and Social Policy and Practice), SCIE Online and Cochrane Reviews and reference searching, with the last search being conducted in April 2021. Fifteen papers were identified as eligible for inclusion. Most of the interventions aimed to improve dementia care (*n* = 10), with others exploring LGBT+ competency (*n* = 2), or other forms of professional development (*n* = 3). Common features of effective learning included a multifaceted approach, with in‐practice learning being blended with additional implementation strategies (e.g. supervision) and didactic learning/worksheets. An important contextual factor was working within an organisational culture which supported shared learning and reflection. This may also help encourage engagement with training, where staff are unwilling to attend if it may compromise care delivery. Future research should focus on the characteristics of trainers and the structure of learning, with more research being needed in in mental and physical morbidities outside the remit of dementia to improve the overall quality of the social care workforce.

**What is known about this topic?**

- The quality and quantity of the social care workforce is known to be inadequate in the United Kingdom.
- The training available is often quite generic and does not accommodate for more specialist skills and interests.
- Training available tends to be oriented to the needs of people with dementia.

**What this paper adds?**

- There is a need for more in‐practice and multifaceted learning that allows opportunity for structured reflection
- There is a growing body of evidence around the training needs of those supporting the LGBT+ community
- Research into the appropriate characteristics of trainers and appropriate learning structure is needed

## 1 INTRODUCTION

In 2021, it was estimated that there were over 962 million people aged over 60 globally (Age International, 2021). It is anticipated that by 2050 this figure will have risen to 2 billion. Although this rise in life expectancy is welcome, many in this population will have a range of co‐morbidities, increasing the demand for health and social care (NIHR Evidence, 2018).

In response to this, many EU countries have re‐drafted care policies to extend the provision of community‐based long‐term care services. This was to promote effective care for older adults while reducing pressures on informal carers and health services (Hattink et al., 2015). However, this is at a time where there is widespread concern about the available workforce and quality (CQC, 2020; OECD, 2022). Chief among these concerns relate to the availability and suitability of the training the long‐term care workforce receives (Health and Social Care Committee, 2021). Within several OECD countries, the non‐registered long‐term care workforce often do not receive specific training to work within the sector (OECD, 2022). Where they do, the ability of the workforce to retain and embed appropriate skills in practice has been questioned (CQC, 2020).

Between 2020/2021 it was estimated that there were 510,000 direct care (e.g. senior care workers, care workers, community support and outreach workers) jobs in domiciliary care in England (Skills for Care, 2021). As the workforce has expanded, it has brought with it increasing diversity within and across roles; accumulating with it a range of job titles, skills and qualifications (Wilberforce et al., 2017). Non‐registered care staff work in different settings including in people's homes, hospice and residential care (Cavendish, 2013), with roles ranging from home care workers, nursing assistants, support workers, social work assistant and more (Wilberforce et al., 2017). These roles encompass various tasks, such as supporting activities of daily living like bathing, mealtimes or engaging with social activities. It is increasingly clear that such support is often provided under challenging circumstances (Newbould et al., 2021).

Surprisingly, there is no international consensus on the level of degree of learning these support grade staff should be required to undertake, with substantial inter‐country variation (OECD, 2022). In the United Kingdom, availability of vocational training for support‐grade staff has depended on government funding, and a high‐profile review arising from a national care scandal made recommendations for core provision to all such staff (Cavendish, 2013). However, there are ongoing concerns that existing training is not appropriately targeted: either being too high‐level and aimed at qualified staff with clinical training, or else trivially basic around simple ‘awareness’ (CQC, 2020; Herber & Johnston, 2013; Wilberforce et al., 2017), with some services relying on bespoke training being provided in‐house or through private providers (Surr et al., 2017), which may result in high expenditure for the for care companies and mean a lack of oversight on the quality of training provided (Greater Manchester Health and Social Care Partnership, 2017).

Training provision for the homecare workforce lacks a coherent evidence‐base. Despite a body of literature on educational theory (Khalil & Elkhider, 2016), knowing how to best design learning for this staff group is still poorly understood. Existing systematic reviews are often limited in their scope. Commonly, reviews are narrowly focused, such as restricted to the dementia workforce without examining other mental health conditions (Eggenberger et al., 2013; Surr et al., 2017), or are focussed on other specific user needs (Higgins et al., 2019; Jurček et al., 2020). Therefore, opportunities for generalised learning are lost (Smith et al., 2011). An attempt to bring together diffuse literature to explore the training and education needs of homecare workers supporting those with dementia and cancer (Cunningham et al., 2020) omitted the broader non‐registered social care workforce. Further research is therefore needed to assess, synthesise and extrapolate the key components of effective learning for non‐registered practitioners in long‐term care to better inform the development of future learning.

A review of reviews was deemed appropriate to compare and contrast existing reviews in line with their assessed quality, to synthesise different components of learning from a broader range of interventions and identify the best evidence for learning design (Smith et al., 2011). With the findings being synthesised alongside the analysis phase of the Analysis, Design, Development Implementation and Evaluation (ADDIE) framework (Mayfield, 2011).

## 2 METHODS

### 2.1 Aim and research questions

We aimed to identify and synthesise evidence to support the design of learning interventions for non‐registered practitioners supporting older people in long‐term care.

Our objectives were to find evidence on the following five components of learning design:

1. Content
2. Format (teaching strategies and resources/media)
3. Structure
4. Contextual factors (barriers and enablers)
5. Measures used when monitoring the effectiveness of learning

### 2.2 Search strategy

The search strategy (Table S1) was informed by previous similar reviews (Dickinson et al., 2017; Frost et al., 2018; Surr et al., 2017; Wells et al., 2020) and a learning development expert at the Social Care Institute for Excellence (SCIE). Databases searched included Pro‐quest (ASSIA), Scopus, Ovid (PsycINFO, Medline, Embase and Social Policy and Practice), SCIE Online and Cochrane Reviews and reference searching.

Search terms were organised into four groups: non‐registered practitioners (population), learning (intervention), community setting (context) and review terms (method), with NOT ‘child*’ being included and older age filters being selected to further refine the results. Titles and abstracts were searched for keywords and reference lists harvested for further relevant reviews with the search concluding in April 2021.

### 2.3 Eligibility and screening

A review of reviews allowed us to check the consistency of our findings on the efficacy of interventions, with the best‐quality reviews being highlighted and synthesised separately if inconsistencies in conclusions are found (Smith et al., 2011).

Inclusion criteria:

- Review focussed on learning interventions provided to non‐registered practitioners supporting older people
- Settings: people's own homes, hospices or nursing/residential care
- Study designs: systematic reviews, meta‐analysis, narrative reviews, scoping reviews, Cochrane reviews
- Papers synthesised the findings from more than one study
- Papers focus on the link between training components, structure, format and/or delivery mechanism and associated outcome(s)
- Published since 2000
- Any country

Exclusion criteria:

- Focus on training for professionally qualified staff
- Focus on those caring for younger people
- Not in English
- Hospital/clinical settings only
- If it did not collect outcomes data
- Does not focus on learning
- Review of reviews

The papers were imported into Covidence (https://www.covidence.org/) with one researcher (L.N.) screening titles and abstracts. Three randomly selected full‐texts were identified for further review among the research team (L.N., M.W., K.S.) to check for consistency and to refine the eligibility criteria prior to commencing full‐text screening (L.N.).

### 2.4 Quality appraisal

The quality of the included papers were reviewed by one researcher (L.N.) using the AMSTAR 2 checklist. This was chosen as it has been shown to have a high level of inter‐relater reliability and usability in comparison to similar tools (Gates et al., 2020), in addition to its being designed to accommodate reviews of non‐randomised studies (Pieper et al., 2019; Shea et al., 2017).

### 2.5 Data extraction and synthesis

Findings were synthesised using the ADDIE framework; an instructional design model often used to support the development of training courses (Mayfield, 2011; Vejvodová, 2009). The ADDIE framework is comprised of five phases: analysis, design, development, implementation and evaluation (Peterson, 2003; Vejvodová, 2009). The findings are synthesised to inform the analysis phase of learning, creating a picture for overall instructional design.

Data was extracted by one researcher (L.N.) in Excel. Data extracted included information on: author, year, aim of the review, type of review, number of studies included, target group, objectives of interventions, methods of synthesis, successful and unsuccessful intervention components (content, format, structure and objective realisation) and outcomes.

The data was then synthesised to identify the key components of the intervention found to influence the effectiveness of the learning. The components included the following: the content presented, format (media and teaching strategies), structural components (e.g. length of sessions) and environmental factors that may affect the objective realisation of the learning.

### 2.6 Reporting

The review was conducted by the research team in liaison with the advisory group while following PRISMA guidance (Liberati et al., 2009).

## 3 RESULTS

**FIGURE 1:**
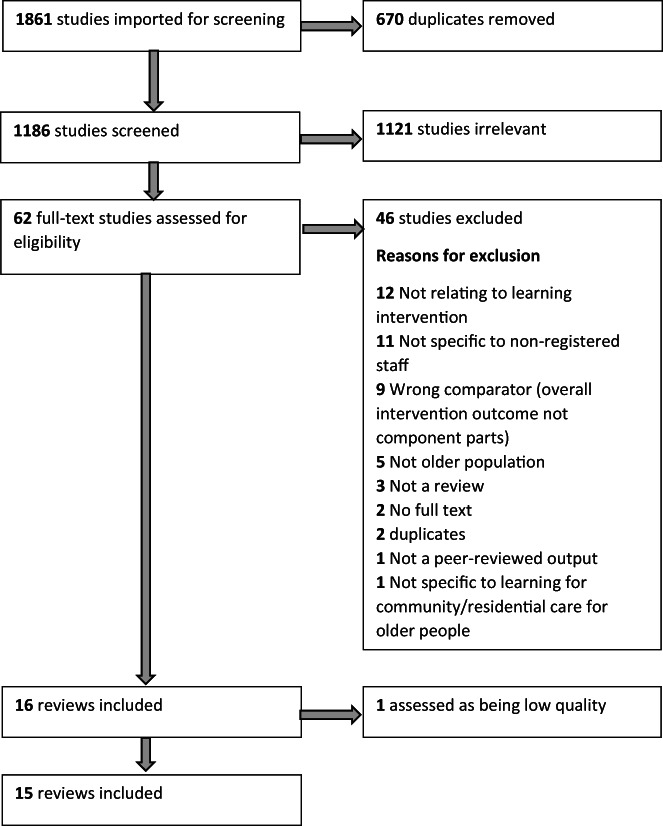
Flow diagram of the review screening process (showing included and excluded reviews)

The flow diagram in Figure 1 shows the screening process, including the reasons for exclusion.

Table 1 shows the PICO components for learning interventions within the included reviews.

**TABLE 1:** The PICO components of learning interventions included within each review

Author	Papers included no.	Synthesis	Population (target group)	Intervention (objective analysis)	Comparators (control vs. intervention)	How did the interventions determine success?
Bauer et al. (2018)	13	Reported in a narrative	Formal caregivers of any clinical background providing care to older adults with dementia in aged care	BPSD and behavioural management skills communication skills; promotion of positive values; raising resident awareness; standardised strategies to promote self‐care and emotion‐orientated care	Most study comparisons were against routine care versus nurse and care staff education	Significance of the results for measures of functional ability and QoL for residents
Cooper et al. (2017)	10	Thematic synthesis	Paid home carers and home care agencies	Sought to improve health or well‐being of older home care clients, and/or staff well‐being	Mainly none or usual care versus specialist dementia home care, activity programmes, medication management, life review therapy or staff training without supervision	Whether the interventions improved the delivery of care, with regard to clients' health and wellbeing and paid carers' well‐being, job satisfaction, and retention
Eggenberger et al. (2013)	12	Qualitative synthesis	Patients with dementia (>65a with a diagnosis of dementia) Healthcare professionals (nurses, doctors, occupational therapists, paraprofessionals) Family caregivers	Enhance communication in dementia care in various care settings	No intervention, wait list group, placebo intervention versus communication skills training or educational intervention in which communication is an essential part	Evaluated if interventions introduced to improve communication in dementia care were effective (changes to behaviour, knowledge skills and attitudes) and safe (adverse effects)
Elliott et al. (2012)	6	Cohen's *d* (where possible) and thematic analysis	Workers who provided support or care to people with dementia and or their informal carers (this could include workers employed in residential facilities or community‐based services)—no papers on community based services were found	Enhance worker capacity and facilitate organisational change	Control (unspecified) or waitlist versus Training interventions with (and without) staff support component	If dementia care training can enhance dementia care worker or workforce capacity such as knowledge of dementia, psychological well‐being, work performance, and organisational factors such as retention or service delivery in dementia care
Higgins et al. (2019)	17	Thematic analysis	Health (nurses, doctors, psychologists, physiotherapists, occupational and speech and language therapists) and social care (social worker or care staff) practitioners	Educational interventions designed to enhance practitioners' competence in relation to older LGBT issues	Identification of pedagogical principles and methods within interventions	Improved education (knowledge, comfort and competence) of LGBT+ issues
Jurček et al. (2020)	9	Qualitative synthesis	Health practitioners (nurses, doctors, psychologists, physiotherapists, occupational and speech and language therapists) social care practitioners (social workers or care staff) or other staff working in aged care services—irrespective of educational level	All interventions aimed to develop cultural competency in relation to LGBT+ older adults by addressing unique issues related to the community	Control group was most often missing	Whether the intervention impacted on knowledge, attitudes and competence of the social care workforce
Kuske et al. (2007)	21	Narrative synthesis	Nursing home staff for institutionalised persons with dementia	To improve dementia care	Control groups (not specified) versus staff training	The interventions' ability to improve staff *knowledge (52%), behavioural changes (48%), attitudes (29%), Caregiver's morbidity or well‐being measured by psychological symptoms, stress levels, self‐esteem, staff turn‐over rates, absenteeism or job satisfaction* Resident outcomes *(rate of inappropriate behaviour (48%), psychological well‐being of residents, the use of psychotropic medications, and the frequency of resident assaults on staff)*
Liao et al. (2019)	8	meta‐aggregative approach	Nursing assistants	Mentors serve as a resource and guide to encourage staff members to develop themselves personally or professionally in an area of importance to them	N/A	Where staff had good experiences of mentorship and were able to successfully implement mentorship programmes
Nguyen et al. (2019)	17	Meta‐analysis and qualitative synthesis	527 residential carers (inc. community): nurses, nursing assistants, nursing aides or recreational/activities officer/coordinators. 267 carers were home‐based/family carers	Communication training programmes that were aimed at improving the regular day‐to‐day interactions between carers and people with dementia	Control group (not specified) versus communication training programmes	Success was determined by the interventions impact on carer *(Knowledge of communication strategies, carer QoL measures)* and care‐receiver outcomes *(QoL or wellbeing mental/physical/social functions and other neuropsychiatric symptoms, such as depression, agitation, disorientation, irritability and withdrawal)*
Pleasant et al. (2020)	19	Narrative synthesis (not stated)	Nine (50%) of the 18 peer‐reviewed publications included just informal caregivers, seven (39%) included just formal caregivers and two (11%) included both formal and informal caregivers	Online training programs. Specifically, dementia‐based online learning, Objectives are not stated, but appear to be orientated to improved knowledge of dementia	Usual care waitlist control, dementia care e‐bulletins, usual care or educational material versus online dementia training	Where online learning to improved knowledge, competency and self‐efficacy and reduced caregiver burden, caregiver stress, depression and anxiety. It also improved care recipient status, and satisfaction among those undertaking the learning
Rapaport et al. (2017)	49	Narrative synthesis	Care home staff	Either interventions that trained care home staff to deliver a specific intervention or that sought to change how care home staff delivered care to residents with dementia	Waitlist control of treatment as usual versus Dementia Care Mapping (DCM), group training without additional supervision	Where the learning is able to improve outcomes for people with dementia in care homes both immediately and long‐term through improved interaction/communication with residents and improved good practice
Rivett et al. (2019)	19	narrative synthesis (not stated)	Staff members working in dementia care homes, nursing homes, assisted living or supported living facilities	Most studies focussed on understanding and managing behavioural and psychological symptoms of dementia (BPSD), with communication/interaction skills being the next most common area of study	Control group not specified versus intervention to improve senses of confidence/competence	Whether the intervention is able to increase the feelings of confidence and competence in social care staff working in long‐term care setting
Spector et al. (2013)	20	Narrative synthesis	Paid care staff in residents living in nursing or residential care homes	To help paid care staff manage BPSD in residents living in nursing or residential care homes	Natural control, waitlist control, or not specified versus training programmes based on different theoretical models	Whether the learning was able to reduce the psychological symptoms of dementia by training care staff to better manage these symptoms
Surr et al. (2017)	152	Critical interpretive synthesis	Staff working in care homes, nurses and nursing assistants/aides	Dementia education	‘no training’ baseline or control versus different training approaches	The ability of the intervention to improve dementia care by improving knowledge, attitudes, confidence, perceived competence, and self‐efficacy in care staff. Thus improving outcomes for people with dementia and their carers
Williams et al. (2016)	76	Realist synthesis	Support workers provide (in clinical or therapeutic settings, community facilities or domiciliary settings)	support worker development interventions	Different bodies of literature (e.g. health, social care, policing and education)	The ability of the intervention to improve skills and care standards of support workers in older people's services

### 3.1 Characteristics of papers

Fifteen papers were included. Seven assessed interventions across a range of care settings (Eggenberger et al., 2013; Elliott et al., 2012; Jurček et al., 2020; Nguyen et al., 2019; Pleasant et al., 2020; Surr et al., 2017; Williams et al., 2016), whereas others were more focused, for example, examining training for staff within residential settings (Bauer et al., 2018; Rivett et al., 2019; Spector et al., 2013) or just nursing homes (Kuske et al., 2007; Liao et al., 2019; Rapaport et al., 2017). Only one paper looked at interventions solely in home care (Cooper et al., 2017), and one did not specify the scope of settings included (Higgins et al., 2019).

Most papers explored the use of training interventions with both non‐registered practitioners and clinical staff, such as nurses (Bauer et al., 2018; Higgins et al., 2019; Jurček et al., 2020; Nguyen et al., 2019; Surr et al., 2017), doctors, psychologists, physiotherapists, occupational and speech and language therapists (Higgins et al., 2019; Jurček et al., 2020), care home staff (Rapaport et al., 2017; Rivett et al., 2019; Spector et al., 2013), both care staff and informal caregivers (Elliott et al., 2012; Pleasant et al., 2020) or nursing home staff (Kuske et al., 2007) and nursing assistants (Kuske et al., 2007; Liao et al., 2019).

The primary objective of five of the reviews was to improve dementia knowledge and care (Elliott et al., 2012; Kuske et al., 2007; Pleasant et al., 2020; Rapaport et al., 2017; Surr et al., 2017). A further three focused on improving the management of behavioural symptoms and improving quality of life (QoL) for older adults with dementia (Bauer et al., 2018; Rivett et al., 2019; Spector et al., 2013). Two papers looked at enhancing communication in dementia care (Eggenberger et al., 2013; Nguyen et al., 2019), another two examined practitioners' competence in LGBT+ issues (Higgins et al., 2019; Jurček et al., 2020). Finally, three studies described professional development support for staff (Cooper et al., 2017; Liao et al., 2019; Williams et al., 2016), for example, mentoring programmes (Liao et al., 2019).

The primary studies within the reviews were mainly conducted within the USA (*n* = 159), UK (*n* = 49), Australia (*n* = 39), Canada (*n* = 37), Netherlands (*n* = 21), Sweden (*n* = 15) Norway (*n* = 13), Germany (*n* = 9), France (*n* = 6), Portugal (*n* = 4), New Zealand (*n* = 3) and Taiwan (*n* = 3). With one research project recruiting participants from both the UK and the Netherlands (Hattink et al., 2015). One review did not provide a breakdown of papers included by country (Williams et al., 2016).

### 3.2 Quality assessment

The reviews were found to be of variable quality (Table S1), with one paper being excluded due to being assessed as very poor quality (Mason & Adeshina, 2011).

Two reviews were rated highly, including Elliott et al. (2012) who reviewed six randomised controlled trials which aimed to enhance dementia workforce capacity, with primary papers being ranked by rigour alongside intervention effect sizes being calculated. The second paper by Nguyen et al. (2019) sought to identify randomised control trials (RCTs), non‐randomised control trials, and controlled before‐and‐after interventions. They pooled estimates for the effects of communication training on carer and care receiver outcomes, with 12 of the papers being identified as RCTs (Nguyen et al., 2019).

The remaining reviews were less qualified in their conclusions but were of moderate quality, with two papers noting the lack of data pertaining to which learning components contributed to successful outcomes (Bauer et al., 2018; Cooper et al., 2017). One noted difficulties in drawing comparisons due to the heterogeneity of interventions (Jurček et al., 2020) and another, methods used within studies (Rapaport et al., 2017); with Elliott et al. noting a bias towards published papers, with unpublished papers being more likely to present a null result (Elliott et al., 2012). Where studies had reported mixed or null findings, these had been attributed to a range of methodological issues (Pleasant et al., 2020). Other quality concerns included poor description of the interventions and strategies for change (Elliott et al., 2012; Rapaport et al., 2017; Surr et al., 2017), such as strategies to address negative attitudes among care staff working with those who identify as LGBT+ (Higgins et al., 2019) as well as poor reporting of study methods (Nguyen et al., 2019; Rapaport et al., 2017) and testing of interventions more generally (Kuske et al., 2007; Spector et al., 2013). Two reviews noted the use of measures that had not been validated to capture outcomes (Rivett et al., 2019; Surr et al., 2017).

Despite the methodological weaknesses identified by review authors, they were able, in most circumstances, to report on the intervention components that were most strongly evidenced in terms of the improved effectiveness (or not) of the learning. These findings are described below.

### 3.3 Content

Content was categorised in broad themes to help understand how information could be best presented within training. Fourteen papers reported relevant aspects of content. One paper was not included in this section as it reviewed the implementation of mentorship programmes, whereby content is personalised to the learner (Liao et al., 2019).

Three papers noted the overall effectiveness of the interventions was improved when the learning was developed using a theoretical framework (Higgins et al., 2019; Spector et al., 2013; Williams et al., 2016) such as, the theory of planned behaviour (Williams et al., 2016). Spector et al. (2013) found that training aimed to reduce behavioural and psychological symptoms in dementia, ‘behavioural‐oriented approaches with person environment fit’ (derived from social theory) and person‐centred care were more likely to be successful when compared to other theoretical approaches, such as emotion‐orientated (drawn from the validation therapy model) and communication approaches (enabling staff to understand how communication can encourage conversation or trigger behaviour), where the evidence is suggested to be weaker and more inconsistent (Spector et al., 2013).

More successful learning was generally orientated to providing practical steps to improve care and set meaningful goals, such as through goal attainment scaling with older adults (Cooper et al., 2017), while ensuring that the teaching was relevant to the practice of the learner (Nguyen et al., 2019; Rivett et al., 2019; Surr et al., 2017; Williams et al., 2016). Sharing of service user information within teams to improve continuity of care was also found to be beneficial (Cooper et al., 2017), in addition to teaching staff reflective practice to encourage independent problem solving (Rivett et al., 2019). Where content lacked relevance to the learner's role, this reduced the effectiveness of the intervention (Surr et al., 2017).

Four reviews found that interventions were more successful when at least some of the content arose from service user engagement throughout the course of the learning and undertaking activities that encouraged self‐refection. Examples included allowing staff to get to know the service user and their experiences better (Cooper et al., 2017; Higgins et al., 2019; Jurček et al., 2020; Rapaport et al., 2017). One way of achieving this was suggested to be through life review with historical accounts of the lives of people with care needs (Cooper et al., 2017; Higgins et al., 2019). This was particularly pertinent when challenging negative attitudes and beliefs (Higgins et al., 2019; Jurček et al., 2020). However, one review noted that interventions promoting emotional and physical closeness sometimes led to learners being fearful of becoming attached to service users (Rapaport et al., 2017).

More generally, equality and diversity content was found to be important. This included training which paid attention to negative institutional factors, legal issues, and protection of marginalised groups (Higgins et al., 2019) to reduce inequalities in care and improve cultural competence of the workforce. One way of achieving this was developing training designed to help social care practitioners to recognise diversity *within* groups of individuals with minority characteristics, for example, LGBT+ older adults (Higgins et al., 2019; Jurček et al., 2020). This was helpful in amending attitudes and beliefs of care staff (Higgins et al., 2019; Jurček et al., 2020).

Providing information on specific caregiver skills was found to be valuable (Pleasant et al., 2020), with the most common skills underpinning care planning and care delivery approaches (Bauer et al., 2018; Cooper et al., 2017; Kuske et al., 2007; Surr et al., 2017), for example, setting meaningful goals with the older adult (Cooper et al., 2017). Specific skills included supporting activities of daily living though improved management of behavioural symptoms (Bauer et al., 2018), understanding the nature of dementia (Rivett et al., 2019; Spector et al., 2013), behaviour management skills (Kuske et al., 2007) and communication skills (Eggenberger et al., 2013; Nguyen et al., 2019). However, one study found inconsistent results regarding the effect of teaching person‐centred approaches and communication skills on the confidence and competence of care staff (Rivett et al., 2019).

### 3.4 Format (teaching strategies and resources/media)

For this section, we combined the media and teaching strategies used to understand what is considered the most effective format for delivery. ‘Media’ encapsulates the range of multimedia used to deliver the training (e.g. video recording, e‐learning, leaflets and manuals). ‘Teaching strategies’ encompassed how the learning would be delivered (e.g. collaborative or experiential learning) and by whom.

#### Delivery methods

It was found that when delivering the learning it was important to include a variety of teaching methods (Bauer et al., 2018; Higgins et al., 2019; Kuske et al., 2007; Nguyen et al., 2019; Rapaport et al., 2017; Spector et al., 2013; Surr et al., 2017), with practice‐based learning being underpinned by theoretical or knowledge‐based content (Surr et al., 2017). The attainment of learning goals was aided by the dissemination of high‐quality accessible learning materials (Kuske et al., 2007; Rapaport et al., 2017; Surr et al., 2017) such as the following:

- remote (video) or in person lectures (Kuske et al., 2007; Nguyen et al., 2019; Surr et al., 2017);
- manuals (Spector et al., 2013; Surr et al., 2017);
- structured assessment and care planning tools (Pleasant et al., 2020; Surr et al., 2017);
- practice guidelines (Pleasant et al., 2020; Surr et al., 2017) or
- worksheets (Pleasant et al., 2020).

These being supplemented with a variety of experiential learning methods (Higgins et al., 2019; Rapaport et al., 2017) or demonstrations of learning material (Bauer et al., 2018; Nguyen et al., 2019) were also beneficial. Solely passive approaches, such as communicating or disseminating information through lectures, written text or video presentations alone (Kuske et al., 2007; Surr et al., 2017) hindered the achievement of learning outcomes.

Six papers further found that even when conditions and resources allow learning (e.g. modified work schedule, practice opportunities, changes to guidance), where a range of approaches were not utilised, learners were not as successful at transferring new knowledge to practice (Bauer et al., 2018; Kuske et al., 2007; Pleasant et al., 2020; Rapaport et al., 2017; Spector et al., 2013); and were less likely to demonstrate a change in attitude, comfort or confidence in delivering care (Higgins et al., 2019). Favourable designs included the use of additional implementation strategies to reinforce the learning (e.g. supervision or feedback), (Kuske et al., 2007; Pleasant et al., 2020; Rapaport et al., 2017; Spector et al., 2013) or drawing on a range of passive, interactive and reinforcing strategies (Bauer et al., 2018; Higgins et al., 2019; Kuske et al., 2007) to support outcomes.

#### Learning activities

Experiential learning included activities such as simulated learning activities, for example, vignettes (Bauer et al., 2018; Nguyen et al., 2019; Rapaport et al., 2017; Surr et al., 2017), role play (Nguyen et al., 2019; Rapaport et al., 2017; Surr et al., 2017), analysis of film interactions (Rapaport et al., 2017), demonstrations within the care environment (Bauer et al., 2018) or experiential learning via in service practice (Surr et al., 2017). However, the importance of these simulated (or in‐service) activities being followed by a structured de‐briefing process was highlighted. It was also found that role‐play could be distressing for learners if they had not yet had the opportunity to build a trusting relationship with the facilitator and other learners. To address this concern, small group teaching could be adopted (Surr et al., 2017). More personalised and interactive activities (e.g. coaching) appeared to also increase the chance of success (Pleasant et al., 2020). Where activities did not accommodate varying levels of experience and education this was found to reduce the effectiveness of the learning (Rapaport et al., 2017). To maximise the opportunities for success, one paper emphasised that the learning should be adaptable to the context of the system, for example, the individuals undertaking the learning, the team and the wider organisational context, such as organisational strategy and priorities (Williams et al., 2016).

Other important facets included learners having the opportunity to share ideas and experiences through face‐to‐face discussion (Higgins et al., 2019; Nguyen et al., 2019; Surr et al., 2017), reflection (Higgins et al., 2019; Rapaport et al., 2017) or the use of formal structures, such as meetings or a database (Rapaport et al., 2017). For example, a database was used among staff at a nursing home using ‘living room theatre activities’ as a way of improving communication with residents (Rapaport et al., 2017; van Haeften‐van Dijk et al., 2015). However, supporting learners through social media instead of messaging applications or the telephone was found to hinder success (Pleasant et al., 2020). The opportunity for shared learning helped facilitate the feeling of ‘journeying together’ and encouraged reflection (Williams et al., 2016) along with the use of personal journals and/or evidence based self‐assessment tools (Higgins et al., 2019).

#### Reinforcing learning: Organisational support

Organisations supporting the ongoing implementation of the learning was found to be important. This could be achieved by providing ongoing supervision (Cooper et al., 2017; Elliott et al., 2012; Pleasant et al., 2020; Rapaport et al., 2017) with a structured de‐briefing process for real‐time interactions (Nguyen et al., 2019). Where observation was incorporated alongside providing feedback and incentives, this was found to help maintain skill over time (Spector et al., 2013). Learners receiving feedback from clinical staff also improved the effectiveness and sustainability of the learning (Eggenberger et al., 2013). Other suggestions included the use of champions for practice change, buddy visits to support learning (Cooper et al., 2017), mentoring (Elliott et al., 2012) and on the spot coaching (Rivett et al., 2019).

An organisation that encourages and supports feedback on real interactions from colleagues, senior and clinical staff, while allowing learner autonomy in the programme (Eggenberger et al., 2013; Kuske et al., 2007; Nguyen et al., 2019; Pleasant et al., 2020) and providing peer support (Kuske et al., 2007) also supported learner outcomes.

Despite this, one high‐quality paper found no consistent evidence of effectiveness for interventions with or without staff support (e.g. through supervision or mentoring; Elliott et al., 2012). However, Elliott et al. (2012) argues that psychological theory still offers a rationale for inclusions of staff support, while highlighting that the papers included in their review only assess organisational outcomes and do not shed light on the support workers response to the interventions included. Monitoring staff performance was also identified as a way of supporting outcomes in one review (Bauer et al., 2018).

#### Trainer characteristics

With regards to trainer characteristics, two papers recommended service users or carers being involved in training (Higgins et al., 2019; Surr et al., 2017) so that they are able to share their stories (in person or via video; Jurček et al., 2020). This was suggested by two reviews to support a change in staff attitudes and beliefs (Higgins et al., 2019; Jurček et al., 2020).

### 3.5 Structure

Opportunities within the learning to demonstrate and practice skills built into the structure (practice‐based learning) were found to be beneficial (Surr et al., 2017), with the need for care workers to feel continually committed to their clients while undertaking training (Elliott et al., 2012). One review concluded that all staff within care homes should be included on the training, with the learning being built into routine care (Rapaport et al., 2017). Further evidence on structure was limited as fewer papers explored this within their reviews.

The literature provided significant evidence on the appropriate duration and intensity of training, albeit with little clear consensus. One review found a lack of evidence between the intensity of the learning and its effectiveness (Spector et al., 2013); and only one paper recommended the use of booster sessions (Eggenberger et al., 2013). The key arguments for appropriate length came from Pleasant et al. (2020) and Surr et al. (2017). Pleasant et al. (2020) found that the connection between trainer and learner was more relevant than length of time spent training, with growth in outcome measures being identified with approximately 1–6 h of online dementia training. Pleasant et al. (2020) argues that the challenge is to strive to make content as succinct, interactive and personalised as possible. Surr et al. (2017) makes a similar argument, stating that effective training is generally >8 h, with this being split into individual sessions of 90 min or more (Surr et al., 2017).

Two papers reported on efforts to motivate attendance, completion and the application of learning to practice. Lottery‐based incentivisation (based on chance) was found to hinder the ability of the learning to achieve the desired change; with certificates, prizes and monetary incentivisation yielding more positive results as it made it more likely that individuals would feel they have a stake in the learning, which encouraged better engagement and participation (Williams et al., 2016). Finally, one review warned that where training required observations and detailed care plans (e.g. when moving from a task focussed to relationship‐centred approach), depending on the organisational context, these learning interventions may be difficult to sustain (Rapaport et al., 2017).

### 3.6 Contextual factors (barriers and enablers)

Some learning benefits were found to be contingent on the context in which staff worked. For staff working in services which were based around what was important to the individual (i.e. working to a ‘needs‐based’ model of care), this allowed staff more time with service users (Cooper et al., 2017; Rapaport et al., 2017), making it likely that the learning would be more effective than those working to a task‐based model of care (Cooper et al., 2017; Surr et al., 2017). Some services were also better at modifying working practice schedules and introducing policy changes (Kuske et al., 2007) to support effective learning. Examples of this include reducing travel times for carers, by geographically aligning staff more closely with clients (Cooper et al., 2017). Additionally, some services aimed to match staff by clients' native language and used careful rostering to facilitate continuity of care (Cooper et al., 2017). Other benefits were achieved by allocating time for training, and working to develop the role of staff through workforce development strategies (such as creating more senior roles) which were bespoke to the service (Williams et al., 2016). Where this training was then reinforced through broader organisational goals, the learning and outcomes were found to be more sustainable (Williams et al., 2016).

Three papers highlighted the importance of resources. These included: time to learn (Elliott et al., 2012; Kuske et al., 2007; Liao et al., 2019; Surr et al., 2017), for example where it affected opportunities for training and supervision (Rapaport et al., 2017); having supportive mentors (Surr et al., 2017); and the organisation's ability to audit staff performance (Bauer et al., 2018). Similarly, being able to deliver good quality care was found to be directly linked to how satisfied care workers were in their role, with those who had greater job satisfaction being more likely to benefit from training (Elliott et al., 2012). However, concerns over care workers' familiarity with clients' needs was an issue when arranging cover to attend training (Elliott et al., 2012). Additional issues included pressurised shifts when struggling with poor service user engagement (Rapaport et al., 2017) and high staff turnover (Bauer et al., 2018; Rapaport et al., 2017). Finally, e‐learning resources, although found to be helpful in assisting learning outcomes, were said to be resource intensive if they were done well (Surr et al., 2017), so could prove to be detrimental to organisations without sufficient funding.

Five papers examined the importance of the organisational culture in affecting the objective realisation of the learning (Liao et al., 2019; Rapaport et al., 2017; Rivett et al., 2019; Spector et al., 2013; Surr et al., 2017). Three reviews found that where management staff were pro‐active and supportive, this was conducive to fostering a care culture (Liao et al., 2019; Rivett et al., 2019; Spector et al., 2013) and was seen to facilitate learning; while lack of co‐operation and communication within the team was found to negatively impact on learning outcomes (Liao et al., 2019; Rapaport et al., 2017; Spector et al., 2013). Where staff shared resources (Rapaport et al., 2017) and were in a position to implement change, for example, being able to apply learning in practice consistently (Spector et al., 2013; Surr et al., 2017), this again supported learning outcomes. Where changes were imposed in a top‐down way, this was also seen as a barrier to learning success (Rapaport et al., 2017), as was the organisation facilitating a change parallel to the learning, for example, re‐structuring an IT system (Rapaport et al., 2017).

Staff characteristics were also found to influence learning outcomes, not only in terms of the learner's motivation (Elliott et al., 2012) but also in terms of access to: expert clinical supervision (Surr et al., 2017), suitable mentors (e.g. approachable, dependable, knowledgeable; Liao et al., 2019), trainers who are able to create a comfortable environment (Surr et al., 2017), or appropriate on‐site support (Rapaport et al., 2017). Where available, they were seen as being extremely beneficial to learning (Liao et al., 2019; Rapaport et al., 2017; Surr et al., 2017). However, inappropriate staffing ratios were found to be a barrier to supervision (Rapaport et al., 2017). In addition, where there was reduced access to speciality staff, having to bring in speciality trainers was suggested to reduce the sustainability of the learning, due to cost (Bauer et al., 2018). Other barriers included staff feeling unable to develop trusting relationships and mentors not being engaged (Surr et al., 2017) further reducing the effectiveness of the learning (Liao et al., 2019). Although opportunities to participate in mentorship were said to be valued, if the mentor was not appropriate, this was found to result in a reluctance to learn (Liao et al., 2019). Inappropriate mentors, included those who are the same grade as the mentee, or where the mentors' role was poorly defined or there was a lack of accountability (Liao et al., 2019). With one paper citing a general lack of willingness to communicate and support each other as a barrier, however, formal mentorship (from mentors who were trained as part of an intervention) was found to address this (Rapaport et al., 2017). Therefore, mentors should have standardised training to support the attainment of outcomes (Liao et al., 2019).

### 3.7 Measures used when monitoring the effectiveness of learning

All the papers included in this review sought to improve long‐term delivery of care, with 10 papers measuring the impact of learning at six (Bauer et al., 2018; Elliott et al., 2012; Kuske et al., 2007; Nguyen et al., 2019; Pleasant et al., 2020; Spector et al., 2013), seven (Rivett et al., 2019), nine (Eggenberger et al., 2013; Rapaport et al., 2017) or 12 months post‐training delivery (Cooper et al., 2017).

The broadest range of outcomes measured were those for paid and family carers. Not surprisingly, the most commonly captured appeared to be measures of knowledge; primarily to assess improvements in knowledge of dementia care (Elliott et al., 2012; Kuske et al., 2007) and knowledge of strategies to enhance communication in dementia care (Eggenberger et al., 2013; Nguyen et al., 2019; Pleasant et al., 2020; Rapaport et al., 2017; Surr et al., 2017). Two papers also aimed to improve knowledge of issues related to the older adult LGBT+ community (Higgins et al., 2019; Jurček et al., 2020).

Secondary to improved knowledge, changes to behaviour were also assessed (Eggenberger et al., 2013; Kuske et al., 2007), including changes in attitude in dementia care (Eggenberger et al., 2013; Kuske et al., 2007; Surr et al., 2017) and in those caring for people within the LGBT+ community (Jurček et al., 2020). Additional outcomes included improved comfort (Higgins et al., 2019), confidence, competence (Higgins et al., 2019; Rivett et al., 2019; Surr et al., 2017) and self‐efficacy in delivering care (Pleasant et al., 2020; Surr et al., 2017). For informal carers, caregiver burden was also assessed (Pleasant et al., 2020).

Additional outcomes for staff included job satisfaction (Cooper et al., 2017; Kuske et al., 2007), well‐being (Cooper et al., 2017; Elliott et al., 2012; Kuske et al., 2007), stress levels (Kuske et al., 2007; Pleasant et al., 2020), self‐esteem (Kuske et al., 2007), depression and anxiety (Pleasant et al., 2020). QoL measures were also captured in one study (Nguyen et al., 2019) and care recipient status for informal carers (Pleasant et al., 2020). Secondary to these outcomes, three papers assessed organisational outcomes, which included, staff retention (Cooper et al., 2017; Elliott et al., 2012; Kuske et al., 2007), absenteeism (Kuske et al., 2007) and work performance (Elliott et al., 2012).

Outcomes to assess the impact on older adults were also captured including: assessments of the clients' health and wellbeing (Cooper et al., 2017), the functional ability and QoL of those living with dementia (Bauer et al., 2018; Nguyen et al., 2019), their psychological wellbeing (Kuske et al., 2007; Nguyen et al., 2019) and symptoms (Spector et al., 2013), such as observed changes in inappropriate behaviour, and resident assaults (Kuske et al., 2007). One paper also reviewed the use of psychotropic medications (Kuske et al., 2007).

Finally, four papers explored evaluation outcomes, pertaining to the implementation of the learning as a way of improving interventions. This included one paper collecting data on the mentee experience (Liao et al., 2019). Another assessed the satisfaction of those undertaking online learning on dementia care (Pleasant et al., 2020). A realist evaluation explored context‐mechanism‐outcome configurations which may support the improvement of skills (Williams et al., 2016). One review also ascertained whether the learning was safe from adverse effects (Eggenberger et al., 2013).

## 4 DISCUSSION

Good training is essential for homecare, not only to drive‐up quality but also to promote the welfare and social status of its workers. It is internationally recognised that training requirements in homecare lack rigour (Goh et al., 2022; Leverton et al., 2021). Some evidence links poor training as a characteristic trait of ‘dirty work’ occupations (Hansen, 2016) with the prospect that improved standards of learning may improve recruitment and retention and provide a more positive occupational identity (Clarke & Ravenswood, 2019). There is evidence that well‐designed training can support confidence in homecare working, and even reduce the likelihood of experiencing adverse events and burnout (Harrad & Sulla, 2018). A well‐trained workforce with skills in aged caring are also priorities for recipients of support (Goh et al., 2022). An international agenda has coalesced around the need to bolster training of long‐term care workers, to address the shortfall in skills and improve the quality of care (OECD, 2020).

Yet evidence on the appropriate design and implementation of training lacks a synthesis that would enable their use in practice. The literature, as currently situated, is disparate. This review aimed to identify best evidence for the instructional design of learning interventions for non‐registered practitioners.

Fifteen suitable reviews were identified, with the majority exploring training in a range of different settings, predominantly aiming to improve dementia care, with extremely limited evidence being available to support training for non‐registered staff supporting older adults with other conditions. The reviews were generally moderate quality as measured on the AMSTAR2 checklist. Only two reviews were identified as being high quality meaning that these results, despite drawing on the conclusions of a range of reviews, should be interpreted with caution.

This findings suggest it is pertinent to adopt a multifaceted approach to learning, with opportunities for in‐practice and interactive learning alongside didactic activities to support outcomes. This is supported by Cunningham et al. (2020) who suggest that active learning approaches are considered best practice within education research. Optimum training length was found to be 8 h in total, with each session being ≥90 min in length. However, few reviews considered the reality of delivering this in a homecare context. In England, but also mirrored further afield, homecare is typically tightly rostered due to constrained funding and workforce shortages. Opportunities for releasing staff for prolonged training, outside of that mandated by any regulator, may be challenging to adopt. However, given the importance of the relationship/rapport between the trainer and the learners, which can take time to develop, finding ways to deliver the same benefits in less time may prove impractical.

This study found greater support for face‐to‐face learning over online counterparts. This is contrary to the current trends in training, which is towards distance‐learning through web‐hosted platforms. However, all the included papers were published before the Covid global pandemic. Arguably, working practices during the pandemic have improved our ability to interact online (Local Government Association, 2022; Winters & Patel, 2021). Therefore, questioning the validity of the findings in modern day seems reasonable. Pleasant et al. (2020) previously found the outcomes of online dementia training to be encouraging, but noted the importance of online training being succinct, interactive and personalised. Surr et al. (2017) also found that online training was well received by users due to its perceived flexibility, but if done well likelier to be resource intensive. Online learning opens‐up the idea of more international/national training and greater opportunities for shared learning (Hattink et al., 2015), and, in a post‐covid environment, evidence for online training may be worth re‐examining. In addition to tight schedules and large workloads (Kelleher et al., 2022), this review also identified that care workers were found to be less willing to attend training if they felt unable to do so without compromising the needs of the older adults under their care (Elliott et al., 2012). This is likely to be particularly important in the context of home support, where discontinuities in care are known to cause substantial interruptions to service quality.

Linked to this is concerns over staff wellbeing. During the covid pandemic, the welfare of the care workforce was challenged, and many reported not having the support needed to protect their emotional wellbeing (McFadden et al., 2021; Shembavnekar et al., 2022). Cunningham et al. (2020) noted that training that does not respond to the needs of the workforce, by supporting self‐care and resilience, may exacerbate these difficulties where there is high stress and burnout.

Finally, this review has identified the first cohort of reviews assessing the needs of the older adult LGBT+ community (Higgins et al., 2019; Jurček et al., 2020). This indicates a change in acceptance, while promoting the voices outside the heteronormative population. The presence of review papers devoted to LGBT+ older adults legitimises this area of research as a priority.

### 4.1 Implications for policy and practice

This finding of this review suggests a multifaceted approach, based on theory may be best suited to improving learning outcomes in non‐registered care staff, with training consisting of the following:

- in‐practice learning with opportunities for structured feedback and reflection on real‐life interactions;
- appropriate mentoring and opportunities to share learning within the team;
- opportunities for staff to draw on emotional and wellbeing support when needed; and
- easily obtainable written information (e.g. learning tools).

These recommendations may support learning alongside team leaders facilitating an organisational culture in which learners feel able to feedback and reflect openly without fear of criticism (Edmondson, 2018).

### 4.2 Strengths and limitations

The key strength of reviews of reviews is them allowing the assessment of the best evidence, while validating the conclusions drawn from other work (Smith et al., 2011). This enables more informed decision making in intervention design. However, some of the papers included within these reviews also incorporated a small number of learning interventions from other populations (such as informal carers and professional staff), which may have contaminated the results. Additionally, the learning derived from reviews of reviews are only as good as the papers collated within them (Smith et al., 2011). The quality assessment was also only conducted by one reviewer, others may have rated these papers differently.

A key strength of this review is the application of the ADDIE framework for the synthesis of the findings, enabling a more systematic and comprehensive analysis of the relevant aspects of learning design for the non‐registered workforce in long‐term care. It also improves the generalisability of the learning from the included studies (Peterson, 2003). However, synthesising across conditions may have some limitations, for example some learning components identified may be condition specific.

## 5 CONCLUSION

Only two high‐quality reviews were identified, with most exploring learning to support the dementia care workforce. In terms of instructional design of the learning, evidence was most readily available on the format of the learning, with less being available on structure and characteristics of appropriate trainers. There is still a scarcity of information on how individual learning components impact on outcomes, with this review only being able to provide broad recommendations for practice. There is a need for further research on the effectiveness of the individual components of learning in achieving outcomes and the development of learning for non‐registered practitioners supporting older adults with a range of morbidities outside the remit of dementia, to better understand how wider training can improve the delivery of care more generally.

## AUTHOR CONTRIBUTIONS

Louise Newbould undertook all data extraction, analyses and drafted the paper. All other authors contributed to (1) developing the aims and methods used in the review; (2) conducting cross‐coder analysis (3) further drafts and revisions. All authors read and agreed on the content of the final submitted manuscript.

## FUNDING INFORMATION

This study is funded by the National Institute for Health Research (NIHR; 102645/CM/UYYB‐P171) School for Social Care Research (SSCR). The views expressed are those of the authors and not necessarily those of the NIHR or the Department of Health and Social Care.

## CONFLICT OF INTEREST

The authors declare no conflicts of interest.

## Supporting information


Table S1.

## Data Availability

Data sharing is not applicable to this article as no new data were created or analysed in this study.
